# The Dark Factor of Personality and Risk-Taking

**DOI:** 10.3390/ijerph18168400

**Published:** 2021-08-09

**Authors:** Shambhavi Tiwari, Morten Moshagen, Benjamin E. Hilbig, Ingo Zettler

**Affiliations:** 1 Department of Psychology, University of Copenhagen, 1353 Copenhagen, Denmark; ingo.zettler@psy.ku.dk; 2 Institute of Psychology and Education, Ulm University, 89081 Ulm, Germany; morten.moshagen@uni-ulm.de; 3 Department of Psychology, University of Koblenz Landau, 76829 Landau, Germany; hilbig@uni-landau.de

**Keywords:** dark factor of personality, dark traits, risk-taking, DOSPERT, behavioral risk-taking

## Abstract

Aversive personality traits have been linked to risk-taking across various domains. Herein, we investigated whether the common core of aversive traits, the Dark Factor of Personality (D), is related to risk-taking. Whereas the conceptualizations of D (common core of aversive traits) and risk-taking (not inherently socially and/or ethically aversive) do not necessarily imply an association, several theoretical considerations do suggest a positive relation between the constructs. In three studies (overall *n* = 689), we linked D to various self-report measures of risk-taking (Studies 1 and 2), as well as to a behavioral risk-taking task (Study 3). Overall, D was positively (although not always statistically significantly) related to self-reported risk-taking in terms of financial, health-related, and recreational risk-taking, fearlessness, novelty sensation seeking, intensity sensation seeking, and drug use. However, we did not find an association between D and behavioral risk-taking. Our findings provide insights into the relation between aversive personality and risk-taking, but also point to inconsistencies depending on the specific nature of risk-taking studied.

## 1. Introduction

Risk-taking can be defined as engaging in activities considering the likelihood of potential desirable and undesirable outcomes [1,2,3,4]. Several aspects around risk-taking have been studied, including risk perceptions (i.e., people’s judgments and evaluations about risks they might be exposed to [5]), risk preferences or attitudes (i.e., the extent to which people are willing to take on risk [6]), and actual risk-taking behavior in terms of decision-making and behavior under uncertainty [7]. 

Theories aiming to explain risk-taking have focused on contrasting computational and rational decision-making models with systematic deviations in actual behavior [8,9]. Psychologists and other decision-making researchers have particularly investigated the role of individual-level and social factors in this regard [10,11,12]. Concerning individual-level factors, variables, such as age, gender, income, wealth, or cognitive abilities have extensively been considered as predictors or correlates of risk preferences and risk-taking behavior [13,14,15]. For instance, it has been found that risk-taking typically decreases in adulthood [16,17], and that men typically show more risk-taking than women, although this might differ depending on the context of risk-taking [18,19]. Moreover, personality characteristics such as impulsivity [20,21,22,23], sensation seeking [23,24,25], or tolerance to ambiguity [26,27] are positively linked to risk-taking, whereas characteristics such as anxiety [28] show negative links.

Among other personality characteristics, aversive (often called “dark”) personality traits—defined as subclinical stable dispositions related to socially and/or ethically aversive behavior [29,30]—have also been studied with respect to their implication for risk-taking. Examples span across risk-taking in several domains including financial [31,32], health and safety-related [33,34,35], lifestyle-related [36,37,38], and risk-taking in a social domain, e.g., concerning dealing with other people [23]. For instance, Psychopathy, a trait typically associated with an erratic lifestyle, impulsivity, and a toleration of danger, has been linked to taking needless risks for minimal gain [23,39,40,41]. In addition, Narcissism, variants of which involve beliefs of entitlement and deserving more than others, has been linked to gambling [32,42] and investment risk-taking [43], arguably because of a tendency to downplay the chances of potential losses. Further, Sadism, i.e., causing interpersonal harm for the purpose of enjoyment, has recently been linked to thrill-seeking [44] as well as to financial, health-related, and recreational risk-taking [3].

As several studies have looked at the relations between various specific aversive traits and various forms of risk-taking, research has accumulated a large body of evidence in recent years that different aversive traits overlap conceptually, empirically, and sometimes even operationally (i.e., almost identical items). This overlap has been attributed to one common underlying basic disposition (e.g., [45,46,47]). A comprehensive conceptualization for such a common core was introduced as the Dark Factor of Personality, or simply D [48]. D is defined as “the general tendency to maximize one’s individual utility—disregarding, accepting, or malevolently provoking disutility for others—, accompanied by beliefs that serve as justifications” [48] (p. 657).

Supporting the conceptualization of D, previous studies have shown that one factor representing the commonalities between up to 12 aversive traits—including Egoism, Machiavellianism, Narcissism, Psychopathy, Sadism, or Spitefulness—explains most of the common variance in most specific aversive traits and their indicators [29,48], and that this factor (D) longitudinally predicts levels in aversive traits as well as changes in aversive traits [49]. Further, D has been found to predict self-reported antagonistic, malevolent, and socially aversive outcomes including aggression, criminality, internet trolling, or stereotyping sexualized behaviors [48,50], and actual behavior in terms of cheating or selfishness [29,48,50]. In addition, an inherent aspect of D is that individuals hold “beliefs that serve as justifications” (p. 657), and D has correspondingly been linked to different beliefs, attitudes and worldviews [50]. Although corresponding research is just in its beginning, theorizing suggests that the development of D as a stable disposition is driven by mechanisms similar to the mechanisms driving the development of more specific aversive traits, i.e., genetic, environmental factors, and their interaction [51,52,53]. Indeed, a first study found support for shared genetic and environmental effects for the development of the common core of the dark triad, i.e., Machiavellianism, Narcissism, and Psychopathy (with some differences controlling for age and sex) [54].

Given that D has been established as the common core of aversive traits and because some aversive traits have been linked to risk-taking in previous research, one may ask how D—i.e., the basic disposition underlying aversive traits—relates to risk-taking. That is, it remains entirely open whether people’s core aversive tendencies relate to risk-taking, or whether the empirically found links between aversive traits and risk-taking are rather due to unique, virtually non-aversive features of aversive traits (such as disinhibition in Psychopathy) beyond D. More precisely, on the level of theoretical definitions, D and risk-taking are clearly distinct constructs. That is, D is defined as the tendency of utility maximization at the costs of others (accompanied by justifying beliefs), which does not necessarily or inherently imply more or less risk-taking. In turn, risk-taking per se is not necessarily socially and/or ethically aversive, and therefore does not fall immediately within the theoretical scope of D. Addressing the issue whether D relates to risk-taking thus provides further insights into the nature of risk-taking in terms of how strongly it is linked to aversive personality (and not only to non-aversive features of specific aversive personality traits). Vice versa, it provides further insights into the consequences of aversive personality for an essentially non-aversive outcome.

Even though D and risk-taking are clearly distinct constructs, there are several reasons that do suggest that individuals with elevated levels of D might also exhibit more risk-taking. First, in striving for utility maximization, individuals higher in D might have a stronger tendency to strive for extreme gains, because these come with surplus utilities, such as renown, a particularly high (economic and/or social) status or setting oneself apart in a competitive sense. Additionally, those high in D are likely to believe that they deserve extreme gains or seek extreme gains as a form of confirming their elevated levels of grandiosity and entitlement. Extreme gains, in turn, necessarily occur with small probabilities [55,56] and thus inherently require taking larger risks (e.g., as operationalized by the “coefficient of variation”, computed as the standard deviation of all possible outcomes divided by the expected value; [57]). Thus, individuals high in D may be more risk-seeking because they particularly seek the surplus utilities of extreme gains beyond mere expected values.

Second and relatedly, because people high in D hold beliefs involving own greatness and entitlement, they may be more prone to overconfidence and similar self-serving biases distorting their risk-sensitivity. In simple terms, grandiose self-views may arguably foster the belief that one’s chances of a gain are higher than its given probability (and vice versa for losses). Thus, people high in D might show more risk-taking behavior because their perspective of how likely it is to win and lose, respectively, might be biased due to beliefs of greatness and entitlement.

Third, the increased willingness of individuals high in D to disregard, accept, or even provoke disutility for others arguably implies a certain level of risk-taking. More specifically, by causing disutility for others, people high in D may more often risk negative consequences such as revenge or sanctions, thus suggesting an elevated tolerance for risks. Indeed, in many situations, aversive behaviors imply larger potential gains (as compared to non-aversive behaviors), but concurrently an increased chance of losses [58].

Finally, and related to the third aspect, the risk of losses (e.g., sanctions) incurred due to aversive behavior might—in and of itself—involve added utility in the form of thrill, excitement, or the like. Essentially, the very possibility of costs or sanctions may have utility in the sense of thrill-seeking, which has been repeatedly linked to risk-taking [59,60]. In support of this view, note that D has been found to subsume a large proportion of the variance in Spitefulness [29,48], a trait representing a preference for harming oneself a little for the sake of causing suffering in others [61]. 

### The Present Research

Overall, although the conceptualizations of D and risk-taking do not suggest an inherent link between the constructs, several theoretical considerations do suggest that D might positively relate to risk-taking. Consequently, we investigated the relation between D and risk-taking across different domains. Specifically, we linked D to various self-report measures of risk-taking (Studies 1 and 2) as well as to a widely used experimental, behavioral risk-elicitation task (Study 3). Whereas Study 1 was an ad hoc study, Studies 2 and 3 represent well-powered, pre-registered studies. The pre-registrations (blinded for review) can be found here:

Study 2: https://osf.io/6v2me/?view_only=16da5aaba20c47a5a47465b1dd571ede (accessed on 3 January 2021);

Study 3: https://osf.io/c3y5v?view_only=9c7b43121ef14a6ba2f045c4f4636a21 (accessed on 11 February 2021).

Generally, we hypothesized that D is positively related with risk-taking. The data files and analysis scripts for Studies 2 and 3 can be found on the Open Science Framework (OSF, link: https://osf.io/q7mkt/?view_only=25624f7511694e3b8629eb40d028ba99 (accessed on 28 June 2021); blinded for review).

## 2. Materials and Methods for Study 1

### 2.1. Procedure and Participants

Participants for this online study conducted in German were recruited via a convenience sample. All participants were German and above 18 years old. The sample comprised 99 participants (59 females, 39 males, one ‘other’), aged from 18 to 78 (*M* = 35.84, *SD* = 14.46) years. Participants reported different levels of proficiency in German (95.95% indicated being native, 3.03% being fluent, and 1.01% having good proficiency). 

When entering the study, participants were presented with basic information about the study, asked for consent, and finally asked to provide demographic information about their age, gender, and proficiency in German. Next, participants were asked to respond to one questionnaire assessing D and then to a series of questionnaires related to risk-taking, namely, assessing domain-specific risk-taking, fearlessness, sensation seeking, and drug use. The order of the different risk-taking measures, as well as the order of the items within each measure were randomized. Finally, participants were thanked for their participation and debriefed about the purpose of the study.

### 2.2. Measures 

Participants’ levels in D were assessed via an ad hoc measure comprising 22 items, as also used previously [62]. Sample items include “People who mess with me always regret it”, “I have hurt people because I could”, or “I’ll say anything to get what I want”. The response scale ranged from 1 = *strongly disagree* to 5 = *strongly agree*. In line with prior analyses of this measure [62], we created a mean score of this item set as an indicator for D. The internal consistency estimate of this measure was Cronbach’s *α* = 0.87. 

Domain-specific risk-taking was assessed via a version of the DOSPERT scale [63,64]. Specifically, we administered 4 items for each of the following subscales: risk-taking in the financial, health, and recreational domain. Participants were asked to indicate how likely it is that they would engage in a described activity or behavior, using a response scale from 1 = *extremely unlikely* to 5 = *extremely likely*. Exemplary items are “Investing 5% of your annual income in a very speculative stock” (financial), “Driving a car without wearing a seat belt” (health), and “Going down a ski run that is beyond your ability” (recreational). The DOSPERT subscales yielded acceptable to good internal consistency estimates with Cronbach’s *α* = 0.74 for financial, 0.61 for health, and 0.81 for recreational risk-taking. 

We assessed fearlessness via the 8-item fearfulness facet scale, belonging to the Emotionality domain, of the HEXACO-100 [65]. Sample items include “When it comes to physical pain, I am a tough person.”, or “I don’t mind doing jobs that are dangerous”. Again, a 5-point Likert-scale ranging from 1 = *strongly disagree* to 5 = *strongly agree* was used as a response format. Cronbach’s *α* of the Fearlessness scale was 0.79. Herein, items were scored in a way that higher values indicate higher levels of fearlessness.

Sensation seeking was measured via the German version [66] of the Arnett Inventory of Sensation Seeking (AISS) [67], again using a Likert-scale ranging from 1 = *strongly disagree* to 5 = *strongly agree* as response format. The original AISS consists of 12 items, with 5 items representing the novelty subscale and 7 items representing the intensity subscale. Herein, we administered the full novelty subscale as well as the five items with the highest loadings (based on [66]) in the intensity subscale, resulting in 10 items overall. Sample items include “I think it’s fun and exciting to perform or speak before a group” (novelty) and “It would be interesting to see a car accident happen” (intensity). Cronbach’s *α* was 0.65 for the novelty subscale, and 0.55 for the intensity subscale, and thus in a similar range as compared to studies reported by [66]. 

Participants’ drug use was assessed via the Alcohol, Smoking and Substance Involvement Screening Test (ASSIST), developed by the World Health Organization (WHO) [68]. Originally, the ASSIST consists of 8 items that are answered referring to the use of each of various substances including alcohol, amphetamine-type stimulants (ATS), cannabis, cocaine, hallucinogens inhalants, opioids, sedatives and sleeping pills (benzodiazepines), tobacco products, and ‘other’ drugs. Herein, we administered 3 items of the ASSIST to assess the consumption of alcohol, amphetamine, cannabis, cocaine, hallucinogens, inhalants, opioids, and sedatives in the past three months. The three items were “In the past 3 months how often have you used the substances you mentioned?”, “During the past 3 months how often have you failed to do what was normally expected of you because of your use of (drug)?”, and “Has a friend or relative or anyone else ever expressed concern about your use of (drug)?”. The response scale ranged from 0 = *never* to 6 = *daily/almost daily*. We created an overall composite score that showed a Cronbach’s *α* estimate of 0.46.

### 2.3. Results of Study 1 

Means and standard deviations of as well as correlations (including the 95% confidence intervals) between all variables are shown in Table 1. D correlated substantially with financial risk-taking (*r* = 0.28, *p* < 0.01), health-related risk-taking (*r* = 0.33, *p* < 0.001), recreational risk-taking (*r* = 0.44, *p* < 0.001), fearlessness (*r* = 0.39, *p* < 0.001), and novelty sensation seeking (*r* = 0.50, *p* < 0.001). D also correlated, though descriptively weaker, with drug use (*r* = 0.20, *p* < 0.05). Further, D showed a positive, but non-significant relation with intensity sensation seeking (*r* = 0.19, *p* = 0.065).

Next to the correlation analyses, we tested whether D was a predictor for the various risk-taking measures once controlling for the demographic variables, given that D is known to vary by age and gender [69]. To this end, we conducted multiple linear regression analyses (one for each criterion) including age, gender, language proficiency, and D as predictors (see Table 2) (Re-running all analyses without including language proficiency as a predictor in the regression models did not affect the link between D and the criteria in Studies 1–3). Once controlling for the demographic variables, D remained a significant predictor for health-related risk-taking (*β* = 0.23, *p* < 0.05), recreational risk-taking (*β* = 0.25, *p* < 0.05), fearlessness (*β* = 0.39, *p* < 0.001), as well as novelty sensation seeking (*β* = 0.32, *p* < 0.001). In contrast, D no longer significantly predicted financial risk taking (*β* = 0.20, *p* = 0.07), intensity sensation seeking (*β* = 0.14, *p* = 0.22), and drug use (*β* = 0.14, *p* = 0.22). Notably, the amount of explained variance ranged substantially across the analyses (0.02 ≤ *R²adj* ≤ 0.37).

Overall, this study serves as a first toehold that D is positively related with risk-taking. Specifically, D was positively related with all risk-taking measures in all correlation and regression analyses, although the respective analyses did not reach levels of significance in some cases. Clearly, though, the study is limited in terms of its relatively low sample size, so that we replicated it recruiting a larger sample as well as administering a more strongly validated item set to assess D.

## 3. Materials and Methods for Study 2

Study 2 served to replicate and extend the first study using a larger sample and an established measure of D. The study was pre-registered prior to the collection of the data, including the hypotheses that D is positively linked to each of the risk-taking measures.

### 3.1. Power Considerations

In order to determine an appropriate sample size for testing our hypotheses, we conducted an a priori power calculation via G * Power [70] for multiple regression analyses. The sample size calculation for an *R²* deviation from zero, based on a conservative significance level to deal with the problems of multiple testing (*α* = 0.01), under assumption of *f*^2^= 0.02 and power = 0.99, resulted in a suggested sample size of *n* = 203 for one multiple regression (D predicting an outcome). Oversampling by 10%, we aimed to obtain a sample of 224 participants.

### 3.2. Procedure and Participants

The study was conducted using the online survey software formr (www.formr.org (accessed on 3 January 2021) [71] with participants being recruited via the online survey panel provider Prolific Academic (www.prolific.co; accessed on 3 January 2021). All participants were from the United Kingdom (UK), were above 18 years old, and had a Prolific Academic approval rate of minimum 95 to attract more honest and/or diligent participants (see [72]).

We stopped data collection once obtaining a final sample of *n* = 224 participants (156 females, 67 males, one participant ‘other’), aged from 18 to 68 (*M* = 33.03, *SD* = 12.13) years. Participants reported different levels of proficiency in English (92.41% indicated being native, 7.14% being fluent, and 0.44% being good). Participants were paid a flat fee of £1.75 for participation in the study.

The setup of Study 2 was virtually identical to the setup of Study 1. When entering the study, participants were presented with basic information about the study, asked for consent, and asked to provide demographic information concerning their country of residence, age, gender, and level of proficiency in English. Following that, participants filled out the questionnaire assessing their levels in D, and then a series of risk-taking measures. As in Study 1, the order of the risk-taking measures as well as of the items within each measure was randomized. Finally, participants were thanked for their participation and debriefed about the purpose of the study.

### 3.3. Measures

Participants’ levels in D were assessed via the 35-item D35 [29], using a response scale ranging from 1 = *strongly disagree* to 5 = *strongly agree*. Sample items include “Payback needs to be quick and nasty”, “People who mess with me always regret it”, or “If I ever tormented others, I would feel strong remorse”. We created a mean score of the D35 as an indicator for D. The internal consistency estimate of D was Cronbach’s *α* = 0.93.

The same risk-taking measures were administered as in Study 1, with the exception that we did not assess drug use given its low internal consistency estimate in Study 1. That is, we administered 12 items from the DOSPERT scale [63] assessing financial (herein, Cronbach’s α = 0.78), health-related (Cronbach’s α = 0.57), and recreational risk-taking (Cronbach’s α = 0.79), the 8-item fearfulness subscale (Cronbach’s α = 0.85) from the HEXACO-100 [65], and the Arnett Inventory of Sensation Seeking (AISS) [67]. This time, we administered all 12 items from the AISS, i.e., 5 items in the novelty subscale (Cronbach’s α = 0.63) and 7 items in the intensity subscale (Cronbach’s α = 0.50).

### 3.4. Results of Study 2

Means and standard deviations of as well as correlations (including the 95% confidence intervals) between all variables are shown in Table 3. D correlated with all risk-taking measures, including financial risk-taking (*r* =0.24, *p* < 0.001), health-related risk-taking (*r* = 0.40, *p* < 0.001), recreational risk-taking (*r* = 0.31, *p* < 0.001), fearlessness (*r* = 0.33, *p* < 0.001), novelty sensation seeking (*r* = 0.15, *p* < 0.05), and intensity sensation seeking (*r* = 0.37, *p* < 0.001).

As in Study 1, we further tested whether D was a predictor for risk-taking once controlling for the demographic variables. To this end, we again conducted multiple linear regression analyses (one for each criterion) including age, gender, language proficiency, and D as predictors (see Table 4). Once controlling for the demographic variables, D remained a significant predictor for financial (*β* = 0.17, *p* < 0.05), health-related (*β* = 0.38, *p* < 0.001), and recreational risk-taking (*β* = 0.24, *p* < 0.001), fearlessness (*β* = 0.29, *p* < 0.001), as well as intensity sensation seeking (*β* = 0.28, *p* <.001). In contrast, D did not predict novelty sensation seeking (*β* = 0.10, *p* = 0.15) beyond the demographic variables. As in Study 1, the amount of explained variance in the criteria ranged substantially (0.06 ≤ *R²adj* ≤ 0.23).

Overall, the results of Study 2 support the hypothesis that D is positively linked to risk-taking. In a final study, we tested whether this relation holds while using a behavioral risk-elicitation task, instead of self-report measures.

## 4. Materials and Methods for Study 3

In Study 3, we linked D to a widely used behavioral measure of risk-taking, namely, the Bomb Risk Elicitation Task (BRET) [73]. Again, we hypothesized that D will be positively related to risk-taking.

### 4.1. Power Considerations

To determine an appropriate sample size, we conducted an a priori power analyses using G * Power [70] for a multiple regression fixed model. The sample size calculation with an *R*^2^ deviation from zero, using a conservative significance level (α = 0.01) assuming of *f^2^*= 0.10 and a desired power = 0.99, resulted in a required sample size of *n* = 325. Oversampling by 10%, we aimed to obtain a sample of 358 participants.

### 4.2. Procedure and Participants

The study was also conducted using the online survey software formr (accessed on 11 February 2021) [71] recruiting participants via the online survey panel provider Prolific Academic. All participants were from the United Kingdom (UK), were above 18 years old, and had a Prolific Academic approval rate of minimum 95. Participants who participated in Study 2 were not allowed to participate herein.

Data was collected from 366 participants (241 females, 123 males, two participants ‘other’), aged from 18 to 79 (*M* = 35.95, *SD* = 13.38) years. Participants were at different levels of proficiency in the English language (93.98% indicated as being native, 4.64% as being fluent, and 1.36% as having good proficiency; none indicated being ‘sufficient’ only). Participants were paid a base fee of £0.75 for their participation in the study with an opportunity to earn more money based on their performance in the behavioral risk-taking measure.

When entering the study, participants were presented with basic information about the study, asked for consent, and then asked to provide demographic information concerning their country of residence, age, gender, and level of proficiency in English. Following that, participants were asked to fill out the D35 before the behavioral risk-taking measure. Finally, participants were thanked for their participation and debriefed about the purpose of the study.

### 4.3. Measures

We assessed D with the same measure as in Study 2, namely, the 35-item D35 [29] using a response scale ranging from 1 = *strongly disagree* to 5 = *strongly agree*. The internal consistency estimate of D was Cronbach’s *α* = 0.91.

Behavioral risk-taking was assessed using the Bomb Risk Elicitation Task (BRET) [73]. The BRET is a visual real-time risk elicitation task that can be administered as a static or dynamic version. We used the static version in which participants were shown a 10 × 10 matrix with 100 boxes. Ninety-nine boxes contain a reward, while one box contains a mine programmed to explode at the end of the task. After the instructions, participants were asked to choose a number that represented the number of boxes they wanted to collect, starting from the upper left corner of the square. The position of the mine was determined after participants had made their choice, by randomly drawing a number from 1 to 100. Based on the position of the mine, participants could earn additional money. That is, if participants’ chosen number was greater than or equal to the drawn number, they have harvested the box containing the mine, and participants earned nothing (next to their flat fee). In contrast, if their chosen number was smaller than the drawn number, participants did only harvest boxes containing rewards, thus earning additional money to their base fee (namely, £0.02 for each box harvested). In line with previous research, the number of boxes that participants chose to harvest was considered to reflect their level in risk-taking, with higher numbers suggesting a higher level of behavioral risk-taking [73,74].

### 4.4. Results of Study 3

We excluded participants who selected harvesting only 1 box (*n* = 5) or all 100 boxes (*n* = 2) (Rerunning all analyses while including these 7 participants did not affect the findings.). Means and standard deviations of as well as correlations (including the 95% confidence intervals) between all variables are shown in Table 5. As shown therein, D did not correlate with behavioral risk taking (*r* = 0.07, *p* = 0.18). Even though there was no significant correlation between D and behavioral risk-taking, in line with the pre-registration (and for consistency across the studies) we conducted a linear multiple regression analysis with the BRET score as the dependent variable, and age, gender, language proficiency, and D as predictors. As shown in Table 6, D was not a significant predictor for behavioral risk-taking beyond the demographic variables (*β* = 0.17, *p* = 0.65). Overall, and thus opposed to our hypothesis, we did not observe D to significantly predict behavioral risk-taking (in the BRET task).

## 5. General Discussion

The present research tested the relation between the basic dimension underlying aversive traits—the Dark Factor of Personality (D)—and risk-taking across several domains. Although different aversive personality traits have been studied both independently and jointly with regard to risk-taking [42,75], the present study is the first to test whether D, the common core of aversive traits [48], is systematically associated with risk-taking. Specifically, D was linked to domain-specific risk-taking [63,64], fearlessness [65], sensation seeking [66,67], drug use [68], and behavioral risk-taking (BRET) [73].

In Studies 1 and 2, we found positive relations between D and all self-reported risk-taking measures, namely, financial, health-related, and recreational risk-taking, fearlessness, novelty sensation seeking, intensity sensation seeking, and drug use (note that the links did not always reach statistical significance). Although the conceptualizations of D (common core of socially and ethically aversive traits) and risk-taking (not inherently socially/ethically aversive) do not necessarily imply an association between the constructs, the positive associations found might be explained in several, partly intertwined, ways.

People high in D might seek more extreme gains because these often come with particularly high surplus utilities, but seeking extreme gains necessarily involves taking more risks. It might also be that holding beliefs of entitlement and grandiosity (as is inherent in D) biases individuals to overestimate or overweight their chances of potential gains and/or to underestimate or underweight losses, thus driving a positive association between D and risk-taking. Further, aiming for own utility maximization at the costs of others might already imply some levels of risk-taking because harming others might bring some negative consequences for oneself such as sanctions or retaliation, as well as some levels of thrill-seeking (which is linked to risk-taking). Importantly, we did not test any of these explanations against each other, so future research might investigate the mechanisms in more detail.

When controlling for the demographic variables of gender, age, and language proficiency, relations between D and risk-taking remained statistically significant in the well-powered Study 2 for all except one (novelty sensation seeking) variables. Generally speaking, most of the observed effect sizes between D and risk-taking in our Studies 1 and 2 were well in line with meta-analytical effect sizes in trait research [76], reported in reviews across personality and social psychology research (e.g., [77,78]), and found in large-scale studies linking personality traits to life outcomes beyond demographic variables [79].

Importantly, we found a weakly positive, but not statistically significant relation between D and behavioral risk-raking via the BRET task [73]. Previous research in this regard has presented mixed findings. That is, some studies found specific aversive personality traits, but not others, to be associated with behavioral risk-taking, though assessed via different measures than the BRET task. For instance, from the Dark Triad of Personality (Machiavellianism, Narcissism, Psychopathy [80]), only Psychopathy was found to be negatively linked to one, but positively linked to another behavioral impulsivity measure (Delay-Discounting task and Stop task, respectively), as well as Narcissism to be positively related with the Stop task [81]. Further, in a multiple regression analyses, (secondary) Psychopathy and Narcissism was found to be positively linked to different indices of the Balloon Analogue Risk Task (BART), but not (primary) psychopathy and Machiavellianism [2]. On the other hand, in another study [42] from the Dark Triad traits only Machiavellianism was found to be positively associated (and weakly only) with the BART task. In sum, it appears as if only specific aspects of aversive traits are related to (specific aspects of) behavioral risk-taking, and future research might aim for more comprehensive investigations in this regard.

More generally, large scale personality research clearly indicates that relations between personality traits and outcomes drop in size (substantially) once using behavioral measures for the latter [76]. In this way, the non-statistical finding of Study 3 might also be explained by generally relatively low trait-outcome associations once the latter are assessed with behavioral measures.

Importantly, though, the findings of our Study 3 are well in line with cumulative prospect theory and the endowment effect bias [82,83] as participants in our study generally behaved in a risk-averse manner (*M* = 30.61). In a similar vein, our results concerning age and gender across the studies largely mirror previous research. Roughly speaking, we mostly found a decrease of risk-taking with increasing age [16,17,84,85], as well as often men showing more risk-taking than women [19,85]. Overall, this supports the view that our studies and samples are quite comparable to personality and risk-taking research in general, including common limitations (e.g., focus on Western participants).

Beyond the question of our findings in particular, the consistency in risk preferences across methods has been a central question in risk-taking research. Self-report measures of risk-taking are anchored in people’s actual experiences of risks and risk-taking and are thus found to be more consistent in assessing risk-taking as a general, stable trait [86]. Behavioral measures of risk-taking, on the contrary, typically present risk scenarios in the form of choice architecture (in terms of their presentation format, specific instructions, or framing including probability weighting and loss aversion). These measures are considered to elicit behavior in a more state-like pattern with different queries and cues that might, in turn, rather trigger heterogeneity in risk preferences. For instance, in a study comparing six risk elicitation methods to explore the consistency in risk preferences, authors found a large majority of individuals (88%) to switch from risk-averse to risk-seeking or vice versa at least once across methods [87].

## 6. Conclusions

Overall, the present research found significant relations between D and self-reported risk-taking across domains including health-related risk-taking, recreational risk-taking, fearlessness, and intensity sensation seeking beyond age and gender. Our findings suggest that the common core of aversive traits is related to self-reported risk-taking, arguably due to individuals high in D placing a particularly high emphasis on extreme gains, holding beliefs that bias their risk perceptions and sensitivity, accepting the risk of negative consequences tied to harming others, and/or deriving utility (thrill) from the possibility of negative consequences. Whereas research on D so far has largely focused on aversive outcomes (see for e.g., [29,62]), this is one of the first studies showing that D—the common core of aversive personality traits—also affects non-aversive criteria. On the other hand, D was not found to be strongly related to a behavioral risk-taking measure. In line with previous research on similarities and differences between risk measure [81,88,89], this finding further suggests the importance of a multi-perspective view on risk-taking, with some aspects potentially not linked to aversive personality characteristics at all.

## Figures and Tables

**Table 1:** Means, standard deviations, and correlations with 95% confidence intervals (Study 1).

	Variable	*M*	*SD*	1.	2.	3.	4.	5.	6.	7.	8.	9.	10.	11.
1.	Gender (*f* = 0, *m* = 1)	0.41	0.52											
2.	Age	35.84	14.46	0.09 [−0.11, 0.28]										
3.	Language proficiency	0.05	0.26	−0.01 [−0.20, 0.19]	0.12 [−0.08, 0.31]									
4.	D	2.20	0.58	0.39 *** [0.21, 0.55]	−0.26 ** [−0.44, −0.07]	−0.15 [−0.33, 0.05]	[0.87]							
5.	Financial risk-taking	1.86	0.85	0.29 ** [0.10, 0.46]	−0.04 [−0.24, 0.16]	0.02 [−0.18, 0.22]	0.28 ** [0.09, 0.45]	[0.74]						
6.	Health-related risk-taking	2.33	0.86	0.23 * [0.03, 0.41]	−0.20 * [−0.38, −0.00]	−0.08 [−0.27, 0.12]	0.33 *** [0.14, 0.49]	0.40 *** [0.22,0.55]	[0.61]					
7.	Recreational risk-taking	2.38	1.12	0.30 ** [0.11, 0.47]	−0.39 *** [−0.54, −0.20]	−0.10 [−0.29,0.10]	0.44 *** [0.26, 0.59]	0.40 *** [0.22, 0.56]	0.50 *** [0.34, 0.64]	[0.81]				
8.	Fearlessness	3.17	0.74	0.13 [−0.07, 0.32]	−0.13 [−0.31, 0.07]	−0.14 [−0.33, 0.06]	0.39 *** [0.21, 0.55]	0.20 * [0.01, 0.38]	0.32 *** [0.13, 0.49]	0.55 *** [0.39, 0.67]	[0.79]			
9.	Novelty sensation seeking	2.40	0.87	0.28 ** [0.09, 0.45]	−0.47 *** [−0.61, −0.30]	−0.07 [−0.26, 0.13]	0.50 *** [0.33, 0.63]	0.26 *** [0.07, 0.44]	0.43 *** [0.25, 0.58]	0.66 *** [0.53, 0.76]	0.47 *** [0.30, 0.61]	[0.65]		
10.	Intensity sensation seeking	3.56	0.74	0.02 [−0.18, 0.21]	−0.23 * [−0.41, −0.04]	−0.06 [−0.26, 0.14]	0.19 [−0.01, 0.37]	0.09 [0.11, 0.29]	0.28 *** [0.09, 0.45]	0.48 *** [0.32, 0.62]	0.33 *** [0.14, 0.49]	0.39 *** [0.21, 0.55]	[0.55]	
11.	Drug use	1.30	1.13	0.22 * [0.02, 0.40]	−0.01 [−0.21, 0.09]	0.02 [−0.18, 0.21]	0.20 * [0.00, 0.38]	0.27 *** [0.08, 0.44]	0.43 *** [0.25, 0.58]	0.32 *** [0.13, 0.49]	0.16 [−0.04,0.35]	0.15 [−0.05, 0.34]	0.25 * [0.06, 0.43]	[0.46]

Note. *n* = 99. *M* = mean, *SD* = standard deviation, *f* = female, *m* = male, *D* = Dark Factor of Personality. Values in the diagonal are Cronbach’s alpha. Values in square brackets below the correlations indicate the 95% confidence interval for each correlation. * indicates *p* < 0.05, ** indicates *p* < 0.01, *** indicates *p* < 0.001.

**Table 2:** Results of the multiple regression analyses (Study 1).

	Financial Risk-Taking				Health-Related Risk-Taking				Recreational Risk-Taking				Fearlessness			
Predictors	*β*	*SE*	95% CI β	*p*	*β*	*SE*	95% CI β	*p*	*β*	*SE*	95% CI β	*p*	*β*	*SE*	95% CI β	*p*
Gender (*f* = 0, *m* = 1)	0.21	0.17	[−0.01, 0.42]	0.06	0.15	0.17	[−0.06, 0.36]	0.16	0.23	0.2	[0.05, 0.42]	<0.05	−0.02	0.15	[−0.23, 0.18]	0.81
Age	0.01	0.01	[−0.22, 0.19]	0.91	−0.15	0.01	[−0.35, 0.05]	0.14	−0.34	0.01	[−0.52, −0.16]	<0.001	−0.01	0.01	[−0.21, 0.19]	0.92
Language proficiency	0.05	0.31	[−0.14, 0.25]	0.58	−0.02	0.31	[−0.22, 0.17]	0.81	−0.02	0.37	[−0.19, 0.15]	0.81	−0.09	0.26	[−0.28, 0.10]	0.37
D	0.2	0.16	[−0.02, 0.43]	0.07	0.23	0.16	[0.01, 0.45]	<0.05	0.25	0.19	[0.06, 0.45]	<0.05	0.39	0.13	[0.17, 0.61]	<0.001
R²/R²_adj_	0.12/0.08				0.14/0.10				0.32/0.28				0.16/0.13			
	**Novelty Sensation Seeking**				**Intensity Sensation Seeking**				**Drug Use**							
	***β***	***SE***	**95% CI β**	***p***	***β***	***SE***	**95% CI β**	***p***	***β***	***SE***	**95% CI β**	***p***				
Gender (*f* = 0, *m* = 1)	0.2	0.15	[0.02, 0.37]	<0.05	−0.02	0.15	[−0.24, 0.20]	0.84	0.17	0.24	[−0.06, 0.39]	0.14				
Age	−0.4	0.01	[−0.57, 0.23]	<0.001	−0.19	0.01	[−0.40, 0.02]	0.07	0.01	0.01	[−0.20, 0.22]	0.94				
Language proficiency	0.03	0.26	[−0.14, 0.19]	0.75	−0.02	0.28	[−0.22, 0.18]	0.86	0.04	0.42	[−0.16, 0.24]	0.71				
D	0.32	0.13	[0.13, 0.50]	<0.001	0.14	0.14	[−0.09, 0.37]	0.22	0.14	0.22	[−0.09, 0.37]	0.22				
R²/R²_adj_	0.39/0.37				0.07/0.03				0.06/0.02							

Note. *n* = 99. *β* = standardized coefficient; 95% CI *β* = 95% confidence interval of *β; f* = female, *m* = male, *D* = Dark Factor of Personality.

**Table 3:** Means, standard deviations, and correlations with confidence intervals (Study 2).

	Variable	*M*	*SD*	1.	2.	3.	4.	5.	6.	7.	8.	9.	10.
1.	Gender (*f* = 0, *m* = 1)	1.31	0.47										
2.	Age	33.03	12.13	0.03 [−0.11, 0.16]									
3.	Language proficiency	1.08	0.29	0.11 [−0.02, 0.24]	−0.05 [−0.18, 0.08]								
4.	D	1.94	0.51	0.29 *** [0.17, 0.41]	−0.13 [−0.25, 0.01]	0.26 *** [0.13, 0.38]	[0.93]						
5.	Financial risk-taking	1.95	0.90	0.20 ** [0.07, 0.32]	−0.14 * [−0.27, −0.01]	0.11 [−0.02, 0.24]	0.24 *** [0.11, 0.36]	[0.78]					
6.	Health related risk-taking	2.12	0.80	0.17 ** [0.04, 0.30]	−0.18 ** [−0.31, −0.05]	0.01 [−0.12, 0.14]	0.40 *** [0.28, 0.50]	0.40 *** [0.29, 0.51]	[0.57]				
7.	Recreational risk-taking	2.18	1.08	0.20 ** [0.07, 0.32]	−0.28 *** [−0.40, −0.15]	0.06 [−0.07, 0.19]	0.31 *** [0.18, 0.42]	0.40 *** [0.28, 0.50]	0.32 *** [0.19, 0.43]	[0.79]			
8.	Fearlessness	2.79	0.82	0.33 *** [0.21, 0.44]	−0.03 [−0.16, 0.11]	−0.03 [0.16, 0.10]	0.33 *** [0.21, 0.44]	0.31 *** [0.18, 0.42]	0.33 *** [0.21, 0.45]	0.60 *** [0.51, 0.68]	[0.85]		
9.	Novelty sensation seeking	3.19	0.81	0.25 *** [0.13, 0.37]	−0.07 [−0.20, 0.06]	−0.04 [−0.17, 0.09]	0.15 * [0.02, 0.28]	0.38 *** [0.26, 0.49]	0.26 *** [0.14, 0.38]	0.50 *** [0.40, 0.59]	0.46 *** [0.35, 0.56]	[0.63]	
10.	Intensity sensation seeking	2.65	0.74	0.36 *** [0.24, 0.47]	−0.22 *** [−0.34, −0.09]	0.06 [−0.08, 0.19]	0.37 *** [0.25, 0.48]	0.36 *** [0.24, 0.47]	0.47 *** [0.36, 0.57]	0.61 *** [0.52, 0.69]	0.52 *** [0.42, 0.61]	0.51 *** [0.41, 0.60]	[0.66]

Note. *n* = 224. *M* = mean, *SD* = standard deviation, *f* = female, *m* = male, *D* = Dark Factor of Personality. Values in the diagonal are Cronbach’s alpha. Values in square brackets below the correlations indicate the 95% confidence interval for each correlation. * indicates *p* < 0.05, ** indicates *p* < 0.01, *** indicates *p* < 0.001.

**Table 4:** Results of the multiple regression analyses (Study 2).

	Financial Risk-Taking				Health-Related Risk-Taking				Recreational Risk-Taking			
Predictors	β	*SE*	95% CI β	*p*	β	*SE*	95% CI β	*p*	β	*SE*	95% CI β	*p*
Gender (*f* = 0, *m* = 1)	0.15	0.12	[0.02, 0.29]	<0.05	0.08	0.1	[−0.05, 0.20]	0.22	0.13	0.14	[0.01, 0.26]	<0.05
Age	−0.12	0	[−0.25, 0.01]	0.06	−0.14	0	[−0.26, −0.02]	<0.05	−0.25	0.01	[−0.38, −0.13]	<0.001
Language proficiency	0.04	0.21	[−0.09, 0.17]	0.52	−0.11	0.17	[−0.23, 0.02]	0.09	−0.03	0.23	[−0.16, 0.09]	0.59
D	0.17	0.12	[0.03, 0.30]	<0.05	0.38	0.1	[0.25, 0.51]	<0.001	0.24	0.14	[0.11, 0.38]	<0.001
R²/R²_adj_	0.09/0.08				0.18/0.17				0.17/0.15			
	**Fearlessness**				**Novelty Sensation Seeking**				**Intensity Sensation Seeking**			
	**β**	***SE***	**95% CI β**	***p***	**β**	***SE***	**95% CI β**	***p***	**β**	***SE***	**95% CI β**	***p***
Gender (*f* = 0, *m* = 1)	0.26	0.11	[0.14, 0.39]	<0.001	0.24	0.11	[0.10, 0.37]	<0.001	0.29	0.09	[0.17, 0.41]	<0.001
Age	0	0	[−0.12, 0.12]	0.95	−0.07	0	[−0.20, 0.06]	0.26	−0.19	0	[−0.31, −0.07]	<0.01
Language proficiency	−0.13	0.18	[−0.26, −0.01]	<0.05	−0.1	0.18	[−0.23, 0.03]	0.14	−0.06	0.15	[−0.18, 0.06]	0.32
D	0.29	0.11	[0.16, 0.42]	<0.001	0.1	0.11	[−0.04, 0.24]	0.15	0.28	0.09	[0.15, 0.40]	<0.001
R²/R²_adj_	0.18/0.17				0.08/0.06				0.25/0.23			

Note. *n* = 224. *β* = standardized coefficient; 95% CI *β* = 95% confidence interval of *β*; *f* = female, *m* = male, *D* = Dark Factor of Personality.

**Table 5:** Means, standard deviations, and correlations with confidence intervals (Study 3).

	Variable	*M*	*SD*	1.	2.	3.	4.
1.	Gender (*f* = 0, *m* = 1)	1.35	0.49				
2.	Age	35.94	13.41	0.05 [−0.06, 0.15]			
3.	Language proficiency	1.07	0.31	−0.02 [−0.12, 0.09]	0.03 [−0.08, 0.13]		
4.	D	1.95	0.49	0.24 *** [0.14, 0.34]	−0.20 *** [−0.29, −0.10]	0.11 * [0.00, 0.21]	
5.	Risk-taking	30.61	20.97	0.16 *** [0.06, 0.26]	−0.07 [−0.17, 0.04]	−0.06 [−0.16, 0.05]	0.07 [−0.03, 0.17]

Note. *n* = 359. *M* = mean, *SD* = standard deviation, *f* = female, *m* = male, *D* = Dark Factor of Personality. Values in square brackets below the correlations indicate the 95% confidence interval for each correlation. * indicates *p* < 0.05 and *** indicates *p* < 0.001.

**Table 6:** Results of the multiple regression analysis predicting behavioral risk-taking (Study 3).

	Behavioral Risk-Taking			
Predictor	β	*SE*	95% CI β	*p*
Gender (*f* = 0, *m* = 1)	0.16	2.32	[0.05, 0.26]	<0.01
Age	−0.07	0.08	[−0.17, 0.04]	0.196
Language proficiency	−0.05	3.57	[−0.16, 0.05]	0.313
D	0.02	2.39	[−0.08, 0.13]	0.658
R²/R²_adj_	0.03/0.02			

Note: *n* = 359. *β* = standardized coefficient; 95% CI *β* = 95% confidence interval of *β; f* = female, *m* = male. *D* = Dark Factor of Personality.

## Data Availability

The data presented in this study are openly available on the Open Science Framework (OSF) at https://osf.io/q7mkt/?view_only=25624f7511694e3b8629eb40d028ba99 (accessed on 3 January 2021).
